# Hematopoietic progenitor cell liabilities and alarmins S100A8/A9‐related inflammaging associate with frailty and predict poor cardiovascular outcomes in older adults

**DOI:** 10.1111/acel.13545

**Published:** 2022-02-15

**Authors:** Benedetta Maria Bonora, Maria Teresa Palano, Gianluca Testa, Gian Paolo Fadini, Elena Sangalli, Fabiana Madotto, Giuseppe Persico, Francesca Casciaro, Rosa Vono, Ornella Colpani, Francesco Scavello, Roberta Cappellari, Pasquale Abete, Patrizia Orlando, Franco Carnelli, Andrea Giovanni Berardi, Stefano De Servi, Angela Raucci, Marco Giorgio, Paolo Madeddu, Gaia Spinetti

**Affiliations:** ^1^ Department of Medicine University of Padova Padua Italy; ^2^ IRCCS MultiMedica Milan Italy; ^3^ Department of Medicine and Health Sciences “Vincenzo Tiberio” University of Molise Campobasso Italy; ^4^ European Institute of Oncology (IEO) Milan Italy; ^5^ Department of Biomedical Sciences University of Padova Padua Italy; ^6^ Unit of Experimental Cardio‐Oncology and Cardiovascular Aging Centro Cardiologico Monzino‐IRCCS Milan Italy; ^7^ Department of Translational Medical Sciences University of Naples Federico II Naples Italy; ^8^ Experimental Cardiovascular Medicine Bristol Medical School: Translational Health Sciences University of Bristol Bristol UK

**Keywords:** alarmins, cardiovascular outcomes, frailty, hematopoietic stem/progenitor cells, inflammaging

## Abstract

Frailty affects the physical, cognitive, and social domains exposing older adults to an increased risk of cardiovascular disease and death. The mechanisms linking frailty and cardiovascular outcomes are mostly unknown. Here, we studied the association of abundance (flow cytometry) and gene expression profile (RNAseq) of stem/progenitor cells (HSPCs) and molecular markers of inflammaging (ELISA) with the cardiorespiratory phenotype and prospective adverse events of individuals classified according to levels of frailty. Two cohorts of older adults were enrolled in the study. In a cohort of pre‐frail 35 individuals (average age: 75 years), a physical frailty score above the median identified subjects with initial alterations in cardiorespiratory function. RNA sequencing revealed S100A8/A9 upregulation in HSPCs from the bone marrow (>10‐fold) and peripheral blood (>200‐fold) of individuals with greater physical frailty. Moreover higher frailty was associated with increased alarmins S100A8/A9 and inflammatory cytokines in peripheral blood. We then studied a cohort of 104 more frail individuals (average age: 81 years) with multidomain health deficits. Reduced levels of circulating HSPCs and increased S100A8/A9 concentrations were independently associated with the frailty index. Remarkably, low HSPCs and high S100A8/A9 simultaneously predicted major adverse cardiovascular events at 1‐year follow‐up after adjustment for age and frailty index. In conclusion, inflammaging characterized by alarmin and pro‐inflammatory cytokines in pre‐frail individuals is mirrored by the pauperization of HSPCs in frail older people with comorbidities. S100A8/A9 is upregulated within HSPCs, identifying a phenotype that associates with poor cardiovascular outcomes.

## Graphical abstract

Frailty affects physical, cognitive, and social domains exposing older adults to adverse health outcomes. High physical frailty with increased S100A8/A9 alarmins level in HSPCs (Hematopoietic Stem/Progenitor cells) and blood identify subjects with early cardiorespiratory dysfunctions. Low HSPCs count and high S100A8/A9 levels in the bloodstream associate with more advanced, multidomain frailty predicting major adverse cardiovascular events (MACE).

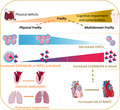

## 1 INTRODUCTION

In the last decades, an unprecedented demographic change has transformed Europe and the world. By 2050, 28.5% of the European population is estimated to be 65 years or older (compared to 20.3% in 2019) with an associated surge in age‐related diseases, including cardiovascular disease (CVD). Thus, the geriatric syndrome of frailty, a state of vulnerability in developing dependency and/or mortality when an individual is exposed to stressors (Clegg et al., 2013), has attracted growing interest (Walston et al., 2019). Identification of frail subjects has become even more relevant during the current COVID‐19 pandemic, which poses major risks in older adults (Hewitt et al., 2020). Two main concepts of frailty have been developed, both proven effective in identifying frail individuals and in predicting outcomes in several clinical settings (Walston et al., 2019). The first, termed “physical frailty,” derives frailty from cumulative declines across multiple physiologic systems, weakness, slowness, weight loss, fatigue, and low activity are used for its assessment (Fried et al., 2001). The second concept ascribes frailty to the accumulation of health, functional, psychological, and social problems (namely “multidimensional frailty”) assessed through the “frailty index” (Searle et al., 2008). In both physical and multidimensional frailty, chronic low‐grade inflammation, alias inflammaging, represents the unifying pathophysiological hallmark, either for the development of sarcopenia and physical exhaustion and for the progression of comorbidities (Ferrucci & Fabbri, 2018). Chronic inflammation is also pathogenically pivotal and therapeutically relevant in CVD (Everett et al., 2019). Among the stress‐secreted factors, the danger‐associated molecular pattern (DAMP) alarmins S100A8 and S100A9 have been associated with cardiac dysfunction (Sallman & List, 2019; Schiopu & Cotoi, 2013; Sreejit et al., 2020). Nonetheless, it remains unknown whether people with signs of physical frailty but still disease‐free, which reportedly represent half of the European citizens aged ≥50 years (Manfredi et al., 2019), exhibit subclinical cardiovascular dysfunction. Nor the existence of shared inflammatory pathways between physical and multidimensional frailty has been explored and tested for an association with clinical outcomes.

Previous investigations of aging mechanisms, including the age‐related deterioration in stem cell functions, provide the basis for new hypotheses about frailty (Schultz & Sinclair, 2016; Sharpless & DePinho, 2007). Bone marrow (BM)‐resident hematopoietic stem/progenitor cells (HSPCs), generally defined by the expression of CD34^+^CD45^dim^, show age‐related changes, with a skewed propensity toward the myeloid lineage (Rossi et al., 2005). A reduction in circulating CD34^+^HSPCs has been associated with the occurrence and severity of CVD (Fadini, 2008; Marvasti et al., 2019). In addition, we and others have shown that, in patients with CVD, HSPCs acquire molecular changes impinging upon their ability to support tissue homeostasis and healing (Dang et al., 2015; Spinetti et al., 2013). It was hypothesized that inflammaging may represent the common denominator of HSPC impairment, frailty, and cardiovascular dysfunction (Ferrucci & Fabbri, 2018; Franceschi et al., 2018), therefore calling for population studies on aging societies.

Herein, we conducted a hypothesis‐generating study to gain preliminary evidence on the molecular and cellular pathways of frailty in two cohorts of elderly people from Northern Italy, one of the most aged European populations. We aimed to determine whether HSPC deficits and molecular marks typical of inflammaging identify a phenotype at risk for accelerated health decline in older people, resulting in cardiovascular complications and death. The relationship between parameters needs a larger confirmatory study in the future.

## 2 RESULTS

### 2.1 Pre‐frail older adults show a subclinical cardiorespiratory impairment

Clinical features of pre‐frail patients are reported in Table 1. The mean age was 75 years, and 57% were males. 66% of patients had hypertension mostly under pharmacological treatment (54%). Cumulative Illness Rating Scale (CIRS) median value was 1.5 (interquartile range [IQR] 1.4–1.6) showing a low chronic illness burden. Average values of blood cell counts, routine laboratory tests, echocardiography, and spirometry were in the normal range (Tables S1–S3).

**TABLE 1:** Clinical characteristics of the pre‐frail cohort

	Pre‐frail cohort (*N* = 35)
Demographics and anthropometrics	
Age (years), mean ± SD	75.0 ± 5.8
Sex male, *n* (%)	20 (57.1)
Body Mass Index (kg/m^2^), mean ± SD	27.9 ± 4.6
Chronic diseases	
Cumulative Illness Rating Scale, median [IQR]	1.5 [1.4–1.6]
Hypertension, *n* (%)	23 (65.7)
Ictus, *n* (%)	1 (2.9)
Coronary artery disease, *n* (%)	1 (2.9)
Diabetes, *n* (%)	5 (14.3)
Diabetic nephropathy, *n* (%)	2 (5.7)
Chronic kidney disease^a^, *n* (%)	1 (3.2)
Pharmacological therapy, *n* (%)	
Oral anti‐diabetics	4 (11.4)
Statins	8 (22.9)
Beta blockers antiarrhythmic	8 (22.9)
Anticoagulants	4 (11.4)
Antiplatelet drugs	14 (40.0)
Antihypertensive drugs	19 (54.3)
Others	26 (74.3)
Frailty	
Italian Frailty index, median [IQR]	9.0 [4.8–11.8]
Physical frailty phenotype, median [IQR]	1.5 [0.5–2.0]
Frailty‐associated questionnaires and tests	
Physical activity scale for the elderly, median [IQR]	74.3 [31.4–110.0]
Mini nutritional assessment, median [IQR]	28.0 [26.0–29.0]
Tinetti scale, median [IQR]	24.0 [19.0–26.0]
6 minutes walking test (m)^b^, mean ± SD	313.1 ± 127.7
Time spent for 4 meters (s)^b^, median [IQR]	4.3 [3.4–6.6]
Muscle strength—Shoulder lift (kg), mean ± SD	10.2 ± 3.4
Muscle strength—Handgrip (kg), mean ± SD	9.4 ± 4.3

Abbreviations: IQR, interquartile range [1st quartile–3rd quartile]; SD, standard deviation.

^a^ For 4 subjects, data were not available.

^b^ For 1 subject, data were not available.

**FIGURE 1:**
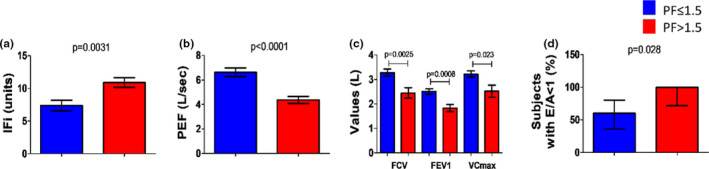
Pre‐frailty status is associated with cardiopulmonary dysfunction. Bar graphs showing the distribution of variables characterizing the pulmonary and cardiac function associated with increased physical frailty (PF). Two groups were identified based on the median PF value of 1.5 (PF ≤ 1.5, *N* = 22, and PF>1.5, *N* = 13). (a) Multidomain frailty expressed by IFi, (b, c) spirometry variables, (d) echocardiography index E/A. Values are means ± SE (a–c) or proportions with related 95% confidence intervals (d)

Due to the low comorbidity burden (CIRS), we used physical frailty phenotype (PF) as a readout of frailty in this cohort. We recorded also Italian Frailty Index (IFi) for comprehensive characterization. Overall, half of pre‐frail subjects reported a PF <1.5 (IQR 0.5–2.0) and IFi <9.0 (IQR 4.8–11.8). Dividing the cohort of subjects into two groups based on the median value of PF (Tables S4 and S5), the group with PF>1.5 showed a higher IFi mean value (10.9 ± 2.7 vs 7.4 ± 3.9, *p* = 0.0031; Figure 1a), and the two indexes of frailty were correlated (Spearman partial correlation coefficient (*r*) 0.54 (95% confidence interval (CI) 0.25–0.75), *p* = 0.0008).

Subjects with PF>1.5 had lower peak expiratory flow (PEF, *p* < 0.0001, Figure 1B), forced vital capacity (FVC, *p* = 0.0025), forced expiration volume (FEV1, *p* = 0.0008), and peak flow rate (VC max, *p* = 0.023) (Figure 1C) (Table S5). Correlation coefficients adjusted for age and sex demonstrated an inverse association between PF and respiratory function measured as PEF (*r* = −0.618; 95% CI = −0.793; −0.349; *p* < 0.0001), FVC (*r* = −0.592; 95%CI = −0.778; −0.321; *p* = 0.0002), FEV1 (*r* = −0.547; 95%CI = −0.749; −0.250; *p* = 0.0008), and VCmax (*r* = −0.463; 95%CI = −0.713; −0.108; *p* = 0.0123) (Table S6).

Regarding echocardiographic parameters, patients with PF>1.5 had a higher prevalence of an E/A ratio <1 (100 vs. 60%, *p* = 0.0280), with a relative risk ratio of 1.67 [95% CI: 1.17–2.38] (Figure 1d). No differences were observed in ejection fraction between groups. In addition, the partial correlation coefficients confirmed a significant negative association between PF and the E/A ratio (*r* = −0.401; 95% CI = −0.669; −0.040; *p* = 0.0304) independently of age and sex (Table S6). Altogether, these data indicate that a higher PF index highlights a subclinical cardiorespiratory impairment.

### 2.2 CD34^+^HSPC count in pre‐frail subjects

We then explored whether pre‐frailty was associated with alterations in BM structure and HSPC count.

By immunostaining, we could not detect differences between subgroups with PF values below or above 1.5, in BM cellularity, that is, density of adipocytes (Figure S1A), vascular profiles (Figure S1B), and myeloperoxidase^+^ hematopoietic cells (Figure S1C). Moreover, no association was found between PF and CD34^+^CD45^dim^HSPC count measured by flow cytometry in the BM or PB (Figure S1D,E).

### 2.3 Pre‐frailty associates with CD34^+^HSPC phenotypical shift and upregulation of S100A8 and S100A9

**FIGURE 2:**
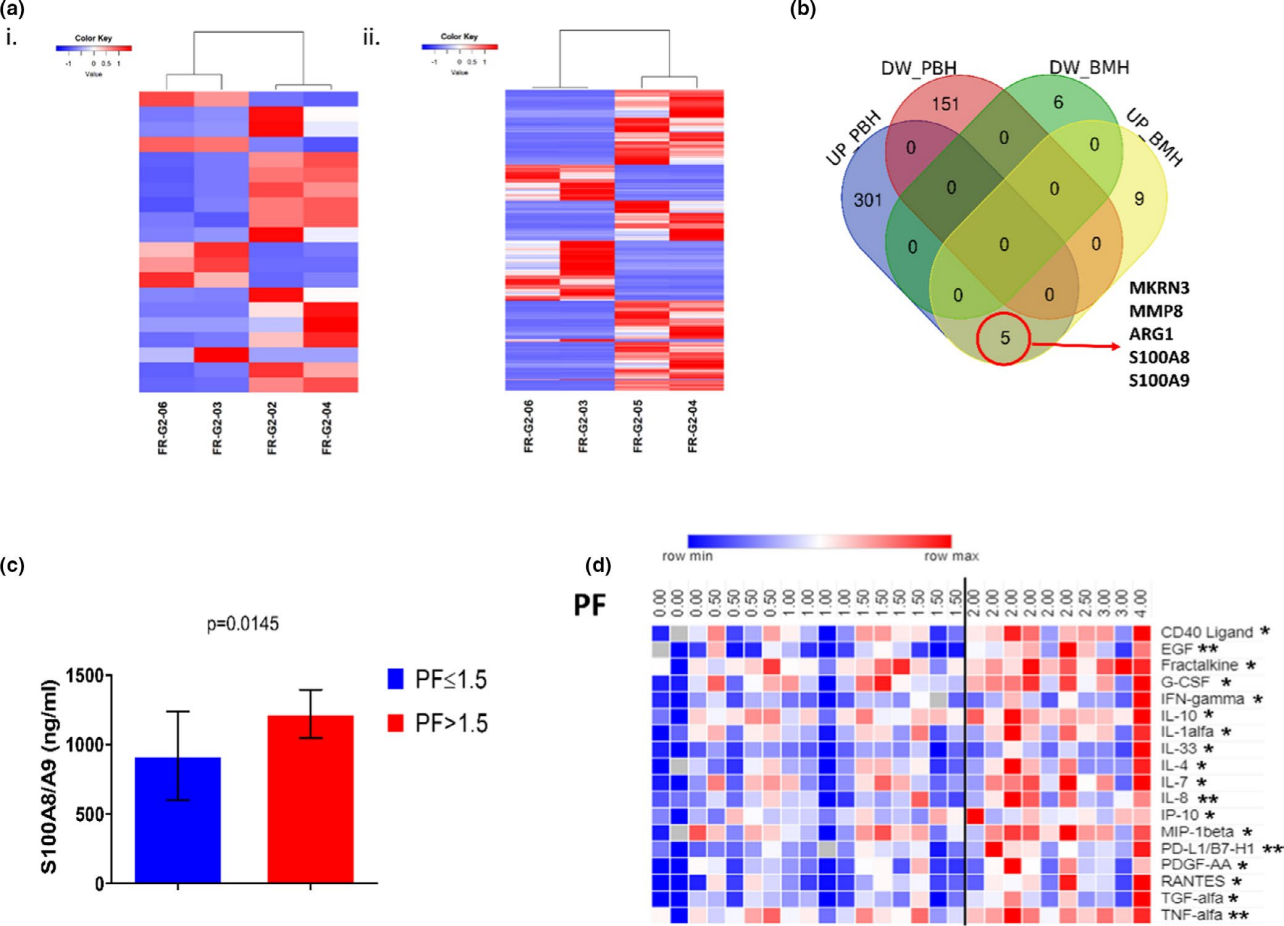
S100A8/A9 high expression levels in CD34+ cells and PB of pre‐frail subjects. (a) RNA sequencing. Heatmaps of modulated transcripts according to CD34^+^ cell sources (i. bone marrow, ii. peripheral blood). (b) Venn diagram with all deregulated genes (UP = upregulated, DW = downregulated, PB = peripheral blood, BM = bone marrow). (c) Levels of the S100A8/A9 heterodimer in peripheral blood measured by ELISA. Mean values ± SE in the two groups stratified according to the median physical frailty (PF) of 1.5. (d) Heatmap of soluble factors quantified by multiplex ELISA assay. Differences between two groups (PF ≤1.5 or >1.5) were analyzed with unpaired two‐tailed Student’s *t* test, * *p* < 0.05 and ** *p* < 0.01

Next, we interrogated the whole transcriptome of CD34^+^HSPCs from subjects with PF≤1.5 or PF>1.5. Principal component analysis (PCA) of RNASeq data showed 1) segregation of BM and PB CD34^+^HSPC samples, and 2) segregation according to the PF for PB CD34^+^HSPCs (Figure S2A), suggesting a specific expression profile of patients with greater PF. These data were confirmed when analyzing BM and PB cells separately (Figure 2a). According to the PF cutoff of 1.5 and cell source, we recognized *MKRN3* (Makorin ring finger protein 3), *MMP8* (Matrix metalloprotease 8), *ARG1* (Arginase1), *S100A8*, and *S100A9* to be the principal differentially regulated hits (Figure 2b). These five transcripts were among the most upregulated in BM‐ and PB‐HSPCs (Tables S7 and S8). Among the differentially expressed genes, subjects with a PF>1.5 displayed a significant upregulation of *S100A8* and *S100A9*. In BM cells, the two transcripts were 13.2‐fold and 7.96‐fold higher compared with subjects with a PF≤1.5. In PB cells, the fold difference was 277 for *S100A8* and 189 for *S100A9*. Furthermore, we performed the ingenuity pathway analysis (IPA) on the differentially expressed genes (DEGs) to identify associated diseases and biological functions (significance expressed as a “z‐score value” equal or superior to |2|). Since the number of DEGs was very low in the BM comparison, we detected no term with a significant z‐score. Instead, the analysis on PB samples showed a multitude of significant terms, including cellular homeostasis, leukocyte number, and biology (Figure S2B).

### 2.4 Upregulation of circulating of S100A8/9 and inflammatory cytokines in pre‐frail subjects

Next, we analyzed the circulating levels of S100A8/A9 and other inflammatory mediators in the pre‐frail cohort. Interestingly, subjects with PF>1.5 had higher plasma concentrations of S100A8/A9 compared with those below (median 1271 ng/ml [IQR 616.5–1962] vs. 513.3 ng/ml [IQR 272.7–701.0], *p* = 0.0145) (Figure 2c). Moreover, subjects with a PF>1.5 displayed a general upregulation of factors including epidermal growth factor (EGF), fractalkine, macrophage inflammatory protein (MIP)‐1β and RANTES, several members of the interleukin family such as IL‐1α, IL‐4, IL‐7, IL‐8, IL‐10, and IL‐33, interferon (IFN)‐γ, transforming growth factor‐alpha (TGF‐α), tumor necrosis factor‐alpha (TNF‐α), and programmed death‐ligand 1 (PDL‐1) (Figure 2d). After considering the effect of confounders (age and sex) in the analysis, the statistical association persisted between physical frailty and S100A8/A9, fractalkine, PDL‐1, RANTES, and TNF‐α (Table S6).

Of note, in the same model, S100A8/A9 inversely correlated with the peak velocity of early diastolic mitral annular motion (index e’) (*r* = −0.405 (95% CI = 0.672; −0.045; *p* = 0.0287), and directly with CRP (*r* = 0.653; 95% CI = 0.376; 0.823; *p* < 0.0001). Altogether, these data suggest HSPC inflammaging is a prominent signature of physical frailty.

### 2.5 Frailty associates with lower HSPC counts and higher S100A8/A9 levels

We further characterize the association between frailty, inflammaging, and cardiovascular outcomes, in a cohort of patients referring to the Division of Metabolic Diseases of the University Hospital of Padova. Baseline clinical characteristics of the 104 enrolled patients that completed the follow‐up are summarized in Table 2. The mean age was 80.6 years, and 54.8% were males. Arterial hypertension and type 2 diabetes were the most prevalent risk factors, while arthrosis, cerebrovascular disease, and chronic kidney disease coronary artery disease were the most common comorbidities. Most patients (92%) were taking four or more medications.

**TABLE 2:** Clinical characteristics of the frail cohort

	Frail cohort (*N* = 104)
Demographics and anthropometrics	
Age (years), mean ± SD	80.6 ± 5.9
Sex male, *n* (%)	57 (54.8)
Body Mass Index (kg/m^2^), mean ± SD	26.8 ± 4.6
Inpatients, *n* (%)	63 (60.6)
Smoking habitude, *n* (%)	6 (5.8)
Chronic diseases	
Charlson Comorbidity Index, mean ± SD	7.5 ± 2.3
Diabetes, *n* (%)	87 (83.7)
Hypertension, *n* (%)	95 (91.3)
Coronary artery disease, *n* (%)	30 (28.8)
Peripheral artery disease, *n* (%)	18 (17.3)
Cerebrovascular disease, *n* (%)	47 (45.2)
Chronic kidney disease, *n* (%)	39 (37.5)
Chronic liver disease, *n* (%)	3 (2.9)
Chronic obstructive pulmonary disease, *n* (%)	11 (10.6)
Osteoporosis, *n* (%)	14 (13.5)
Cancer, *n* (%)	20 (19.2)
Stroke or transient ischemic attack, *n* (%)	11 (10.6)
Arthrosis, *n* (%)	45 (43.3)
Pharmacological therapy, *n* (%)	
Glucose‐lowering medication	83 (79.8)
Angiotensin‐converting enzyme inhibitors or angiotensin receptor blockers	72 (69.2)
Other anti‐hypertensive	77 (74.0)
Statin	62 (59.6)
Antiplatelet	72 (69.2)
Politherapy (>3/day)	96 (92.3)
Frailty	
Italian Frailty index, mean ± SD	14.1 ± 6.9

Abbreviations: IQR, interquartile range [1st quartile–3rd quartile]; SD, standard deviation.

Patients were divided into two groups according to the median value of IFi score (14.1 [IQR = 8.3–18.5]) (Table S9). As expected, the group with IFi>14.1 had a higher Charlson Comorbidity Index (CCI) score. However, while chronic obstructive pulmonary disease was more frequent in those with a higher IFi, no differences were observed in the prevalence of other diseases between the two groups.

In the whole cohort, the mean PB‐derived CD34^+^cell count was 2366 cells/ml (absolute), in line with the levels of CD34^+^ cells/μl described in PB of healthy subjects of the same age (Fukuda et al., 1998). The median level of S100A8/A9 was 853 ng/ml (IQR 518–1301).

**FIGURE 3:**
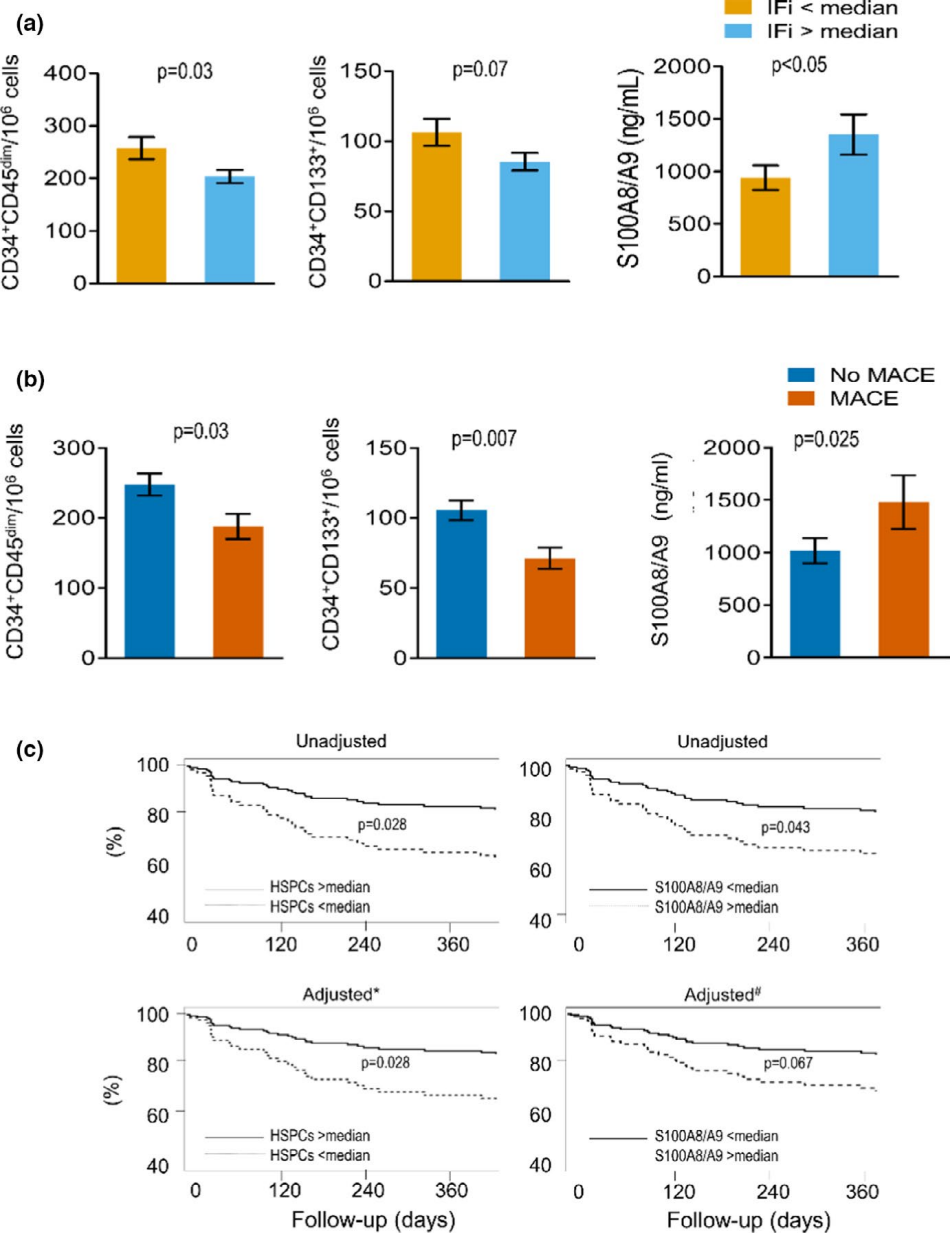
Baseline HSPC and S100A8/A9 levels associates with frailty and predict future MACE. (a) Bar graphs showing the average number of the identified HSPC populations as measured by flow cytometry, and circulating plasma levels of S100A8/A9 in subjects grouped based on the median IFi value of 14.1 (*N* = 52 per group). Values are means ± standard deviation. (b) Bar graphs show the abundance of HSPC phenotypes and S100A8/A9 levels in the two groups. (c) Survival free from MACE curves unadjusted (upper panels) and adjusted for age, frailty index and S100A8/A9 (*) or CD34^+^CD133^+^ HSPCs (#), respectively (lower panels). Significance level (*p*‐values) was assessed through Cox proportional hazard model

Subjects with higher IFi had significantly lower levels of HSPCs identified as CD34^+^ (*p* = 0.025), CD133^+^ (*p* = 0.008), or CD34^+^CD45^dim^ (*p* = 0.030). S100A8/A9 levels were higher in the PB of patients with higher IFi (*p* = 0.045) (Figure 3a and Table S9).

Frailty showed a strong association with circulating S100A8/A9 (*r* = 0.22; *p* = 0.009). Moreover, S100A8/A9 levels were inversely correlated with CD34+ HSPCs, after adjusting for age and sex (Beta = −0.30; *p* = 0.024). Given these multiple inter‐relations, we explored the association between HSPCs (CD34^+^, CD133^+^, or CD34^+^CD45^dim^), in multivariate linear regression analyses adjusted for age and sex with IFi as a dependent variable. IFi was inversely correlated with the HSPC phenotypes independently from age and sex (Table S10).

When the model was adjusted also for S100A8/A9, IFi and CD133^+^ HSPC remained significantly correlated (Table S11 Model 2). In addition, in all the models, IFi and S100A8/A9 remained significantly correlated, independently of age, sex, and HSPC count (Table S11).

### 2.6 HSPCs and S100A8/9 predict cardiovascular outcomes at the 1‐year follow‐up

During the 1‐year follow‐up, we recorded 41 hospital admissions, 21 non‐fatal cardiovascular events (3 non‐fatal stroke, 3 non‐fatal acute myocardial infarction, 13 hospitalizations for heart failure, 2 hospitalizations for unstable angina), and 16 deaths (9 cardiovascular deaths). No patient was lost at follow‐up. Of note, IFi predicted mortality, hospitalization, and Major adverse cardiovascular events (MACE) independently from the CCI (not shown).

Probability to undergo a MACE during follow‐up increased with age (*p* = 0.048), IFi (*p* = 0.001), and CCI (*p* = 0.022) (Table S12). Moreover, patients with incident MACE had lower baseline levels of HSPCs defined as CD34^+^CD133^+^ (*p* = 0.007) and CD34^+^CD45^dim^ (*p* = 0.030), as well as higher levels of S100A8/A9 (*p* = 0.025) (Figure 3b). Reduced CD34^+^HSPC counts and elevated S100A8/A9 levels remained significantly and independently associated with incident MACE after adjusting for age and IFi (Table S13). Figure 3c‐upper panels show unadjusted MACE‐free survival of patients categorized into high/low CD34^+^CD133^+^ HSPCs and S100A8/A9 concentrations. HSPC counts confirmed a prognostic power in an adjusted model, whereas S100A8/A9 showed borderline significance (Figure 3c‐lower panels).

When assessing fatal events at 1‐year follow‐up, IFi (HR 1.11; 95% CI = 1.01–1.22; *p* = 0.024) and S100A8/A9 (HR 2.28; 95% CI = 1.20–4.31; *p* = 0.012) predicted all‐cause mortality independently of confounders (age and cancer) (Table S14).

## 3 DISCUSSION

We report that an inflammaging signature in blood and HSPCs underscores increasing frailty. The signature is already activated in pre‐frail subjects. In frail patients, S100A8/A9 increased concentration is accompanied by depletion of circulating HSPCs, both of which predict the occurrence of MACE.

### 3.1 In pre‐frail older adults, we newly observed a potential contribution of HSPCs to inflammaging and a subclinical cardiorespiratory impairment.

CD34^+^HSPCs represent a heterogeneous cell population with well‐established regenerative capabilities, which is mobilized from the BM after stressor events (Mackie & Losordo, 2011). A low CD34^+^HSPC count in PB has been associated with cardiovascular morbidity and mortality (Fadini et al., 2020), cardiopulmonary syndromes (Liu et al., 2014), and age‐associated diseases (Mandraffino et al., 2017). In addition, HSPCs of patients with CVDs show molecular changes that compromise their homeostatic and healing functions (Dang et al., 2015; Spinetti et al., 2013). Moreover, reduction and molecular derangement of HSPCs predicts cardiovascular mortality in patients with type 2 diabetes (Fadini et al., 2017; Spinetti et al., 2020). In our pre‐frail cohort with increasing frailty, we observed a higher rate of early diastolic dysfunction and impaired respiratory function (i.e., reduced PEF, FCV, FEV1, and VCmax), even in the absence of overt respiratory disease. Nevertheless, HSPC count was not associated with higher PF. Interestingly, although not changed in the count, HSPC showed a PF‐associated transcriptomic shift characterized by increased mRNA transcripts of inflammatory molecules such as alarmins *S100A8* and *S100A9*. Alarmins are expressed by cells of myeloid origin through the differentiation to granulocytes and monocytes, where they represent about 40% and 5% of cytosolic proteins, respectively. They exist as homodimers and associate to form the heterodimer S100A8/A9, known as calprotectin. Intracrine alarmin action results in cytosol tubulin polymerization and cytoskeleton rearrangement, which are instrumental to cell migration. Alarmins can be released from damaged or dying cells, and, by binding to pattern recognition receptors, like Toll‐like receptors (TLRs) and receptor for advanced glycation end products (RAGE), and exert a protective role from infection (Ometto et al., 2017). Conversely, aberrant expression of S100A8/A9 boosts inflammation by accelerating cytokine release by neutrophils and monocytes/macrophages, thus generating a vicious cycle of unresolved tissue damage (Donato et al., 2013; Foell et al., 2007; Wang et al., 2018) through a mechanism that involves NF‐κB induced transcriptional priming (Sallman et al., 2016). In frail subjects, similar to what has been observed in diabetes, excessive S100A8/A9‐mediated RAGE signaling could contribute to myelopoiesis, leukocytosis, and vascular dysfunction (Flynn et al., 2020; Johnson et al., 2021; Nagareddy et al., 2013).

After myocardial ischemia, neutrophils rapidly infiltrate the tissue, starting the inflammatory response needed for tissue repair. By secreting alarmins, neutrophils recruit monocytes that differentiate into cardiac macrophages contributing to the phagocytosis of dead cardiomyocytes via a process defined as efferocytosis (Marinkovic et al., 2019, 2020). In turn, macrophages release cytokines that modulate the inflammatory reaction to allow the formation of collagen fibers instrumental for cardiac repair. Of note, CD150^+^CD48^−^CCR2^+^ HSPCs are involved in post‐myocardial infarction healing (Dutta et al., 2015).

Increased secretion of the heterodimer S100A8/A9 is associated with myocardial necrosis (Schiopu & Cotoi, 2013) and predicts cardiovascular events independently of CRP (Morrow et al., 2008). Of note, elevated circulating levels of S100A8/A9 of myeloid origin reportedly represent a discriminator for COVID‐19 outcome (Silvin et al., 2020), a disease that bears high cardiovascular risk in older people (Hanssen et al., 2021).

In our cohort comprising apparently healthy subjects, higher circulating levels of S100A8/A9 and a signature of inflammatory mediators identified subjects with higher frailty. This signature comprised other DAMP (alarmins, IL‐1α, and IL‐33), senescence‐associated molecules (TNF‐α, EGF, IL‐7, and IL‐8), and the infectious‐triggered cytokine response. Thus, chronic low‐grade inflammation, alias inflammaging, may represent the common denominator of HSPC impairment, frailty, and cardiovascular dysfunction (Ferrucci & Fabbri, 2018; Franceschi et al., 2018).

The source of circulating S100A8/A9, the reason for HSPCs to be caught by inflammaging, and how this can translate into more cardiovascular complications remain undetermined. High levels of plasmatic S100A8/A9 may reflect cell senescence at the systemic or organ level. It can be argued that S100A8/A9‐expressing myeloid cells can transfer their pathogenic cargo to the peripheral vasculature, thereby transforming a healing mechanism into an amplifier of cardiovascular damage. In line with this, we recently reported that microRNA‐21/PDCD4 proapoptotic signaling is conveyed from circulating CD34^+^HSPCs to vascular endothelial cells in patients with critical limb ischemia (Spinetti et al., 2020).

### 3.2 In frail subjects with high comorbidity index, low HSPCs predict MACE, non‐fatal cardiovascular events, and all‐cause hospitalization after adjustment for confounders

In the cohort with physical frailty but no clinical evidence of disease, the two frailty indexes PF and IFi could not identify a difference in the abundance of HSPCs in BM or PB. These data suggest the HSPC deficit takes place after the onset of causative pathological processes on a pre‐frailty condition.

Of note, in the frail cohort, the heterodimer S100A8/A9 predicted all‐cause mortality and MACE independently of confounders. Therefore, we posit that the progression from pre‐frailty to frailty starts from inflammation and then involves HSPCs.

Intriguingly, available drugs exist to block S100A8/A9. Tasquinimod, an oral quinoline‐3‐carboxamide, binds to the S100A8/A9 complex, thereby blocking its interaction with TLR4 or RAGE, inhibiting TNF‐α release (Bjork et al., 2009). Indeed, the compound reportedly exerted therapeutic benefit in inflammatory diseases such as type 1 diabetes (Coutant et al., 1998), SLE (Bengtsson et al., 2012), and multiple sclerosis (Polman et al., 2005); recent studies highlighted the possibility of a therapeutic time window in myocardial ischemia (Marinkovic et al., 2019, 2020).

### 3.3 Study limitations

This observational study was designed as a hypothesis‐generating investigation, and, consequently, the emerging evidence supporting the existence of an association requires further confirmation in larger studies. Subjects enrolled in the pre‐frail cohort suffer from coxarthrosis a degenerative/chronic inflammatory condition that can influence stem cell number and function. Nevertheless, arthrosis is a common disease in older adults and, like other chronic degenerative conditions (Franceschi et al., 2018), can be considered a suitable setting to assess the role of chronic low‐grade inflammation (Cacciatore et al., 2014).

### 3.4 Conclusions

Frailty associated with multimorbidity is a condition at greater risk of adverse outcomes that might benefit from treatment optimization (Turner et al., 2014). While frailty predicts future disability, evidence indicates that this progression might be modifiable, particularly at an early stage, thus improving and prolonging health‐span (Puts et al., 2016). Our study provides novel insight on the cellular and molecular mechanisms of frailty and underscores the importance and practical value of methods assessing both components (disease and loss of physiologic function) of the decline characteristic of aging. In perspective, these new findings provide a new molecular mark of the frailty progression, thereby potentially aiding the prognosis, prevention, and treatment of older frail subjects. This is especially relevant in the context of the COVID‐19 pandemic, considering the importance of tools that may enable clinicians in establishing effective preventive care for selected elderly patient populations.

## 4 EXPERIMENTAL PROCEDURES

### 4.1 Clinical study

In this observational study, we evaluated the relationship between frailty, levels and phenotype of CD34^+^CD45^dim^HSPCs, and circulating markers of inflammaging including S100A8/A9. First, we assessed those variables from BM and peripheral blood (PB) sources in a cohort of 35 pre‐frail individuals characterized for cardiorespiratory function (Figure S3A,B). Next, in 104 more frail subjects, we sought confirmation of the data and studied the relations between circulating markers and outcomes at 1‐year follow‐up (Figure S3C).

#### 4.1.1 Sample size

This was designed as a hypothesis‐generating study, and the group size was decided a priori by the biostatisticians team accordingly. A sample of around 100 subjects, like the one we used for the frail cohort, is standard in hypothesis‐generating studies. Concerning the pre‐frail cohort, we decided to enroll 35 patients because, when the protocol was designed, no information for molecular and cellular pathways associated with physical decline on pre‐frail subjects was available in scientific literature. Given the exploratory nature of the analysis conducted on these subjects, we complied with the recommendation of Lancaster et al. (2004) that suggests a general rule of 30 enrolled patients or greater to estimate a parameter in a “pilot” study.

All patients provided written informed consent to the study, which was conducted following the Declaration of Helsinki and under the ethical approval of the internal ethical Committees (IRCCS MultiMedica protocol #314‐2017 and the University Hospital in Padova protocol #4437/AO/18).

### 4.2 Pre‐frail cohort

A total of 35 consecutive subjects with coxarthrosis referring to MultiMedica Hospital to receive elective hip joint replacement surgery were enrolled between May 2018 and January 2020. We considered this population with chronic low‐grade inflammation susceptible to frailty development and suitable to study CD34^+^HSPCs from both BM and PB.

*Inclusion criteria*. Age >63 years and ability to participate in the evaluation of frailty. *Exclusion criteria*. Participation in other trials, history of coronary artery disease, stroke, clinical conditions expected to impair 2‐year survival, alcohol or drug abuse, and inability to provide informed consent.
*Aim and objectives*. Investigating the relationship between frailty, cardiopulmonary function (assessed by spirometry and echocardiography), and circulating levels of HSPCs and S100A8/A9. In addition, a sub‐group analysis was conducted to determine the molecular signature of BM‐derived CD34^+^HSPCs using RNA sequencing (RNASeq) (Figure S3A).
*Baseline assessment*. Frailty was determined using both the physical frailty phenotype (PF) (Fried et al., 2001) and the IFi (Abete et al., 2017), an adaptation of the Rockwood frailty Score (Searle et al., 2008) (Figure S3B). Moreover, the CIRS was used to quantify patients’ comorbidities (Conwell et al., 1993).
*Examinations performed*. Spirometry, trans‐thoracic echocardiography, and blood sampling for the measurement of biochemical analytes and levels of HSPCs were performed in all the subjects. All patients underwent femoral head replacement surgery for coxarthrosis, and BM samples were collected from surgical leftovers. Reference values for spirometry (Graham et al., 2019) and echocardiography (Lang et al., 2015) were used.

### 4.3 Frail cohort

Patients were consecutively enrolled between November 2017 and March 2019 at the Division of Metabolic Diseases of the University Hospital of Padova.

*Inclusion criteria*. Age >65 years and ability to answer the frailty questionnaire. *Exclusion criteria*. Acute illnesses expected to impair survival within 1 month, current or previous hematological disorders, dementia, or inability to provide informed consent.
*Aim and objectives*. Investigating the associations among frailty, circulating levels of HSPCs and S100A8/A9, and clinical outcomes in older adults. Follow‐up data of the clinical outcome were collected after 1 year by accessing the patients’ electronic medical records and by phone contact when needed.
*Baseline assessment*. Due to comorbidities in this cohort, the frailty status was assessed only using the IFi (Abete et al., 2017). The Charlson comorbidity score was also used to quantify the patients’ comorbidities (Charlson Comorbidity Index, CCI) (Charlson et al., 1987). Blood samples were collected at the time of the IFi assessment for the analysis of HSPCs and S100A8/A9 (Figure S3C).
*Outcomes*. Occurrence of death, hospitalization for any cause, and non‐fatal cardiovascular events (including myocardial infarction, stroke, hospitalization for heart failure, unstable angina and arrhythmia, and unplanned coronary, peripheral, or carotid revascularization). Major adverse cardiovascular events were a composite of death from cardiovascular causes and non‐fatal cardiovascular events.

### 4.4 Physical frailty phenotype (PF)

For PF three or more positive criteria out of the total five parameters recorded to define the subject as frail, 1–2 as pre‐frail, and 0 as robust (Fried et al., 2001). In particular, we registered: 1) unintentional weight loss and 2) exhaustion by a direct question during the visit; 3) low physical activity (sedentary behavior) by using the Physical Activity Scale for the Elderly (PASE) (Curcio et al., 2019); 4) slowness indicating sarcopenia, a hallmark of frailty, by asking the subjects to perform a 6‐min Walking test (6MWT) (Enright, 2003), and 5) weakness by directly measuring muscle strength through the handgrip test (Figure S3B).

In addition, we assessed individual gait and balance by the Tinetti test (Tinetti, 1986).

### 4.5 Italian Frailty Index

It counts deficits in health domains (including symptoms, signs, disabilities, and diseases) (Searle et al., 2008), cognitive function (assessed using the Mini‐Mental State Examination) (Folstein et al., 1975), need of social support (using the Social Support Score) (Mazzella et al., 2010), and nutritional data (using the Mini Nutritional Assessment) (Kaiser et al., 2010). The index is expressed as a ratio of identified deficits to the total number of deficits considered. The iFI scores from 0 to 40 and considers three subgroups of frailty; 0–16.0 mild/light, 16.1–27.0 moderate, and 27.1–40 severe (Abete et al., 2017).

### 4.6 Peripheral blood and bone marrow samples harvesting and processing

From patients enrolled in both cohorts, 20 ml PB was withdrawn by venipuncture and collected in EDTA‐coated tubes for cell analysis using flow cytometry, isolation of CD34^+^HSPC for molecular studies, and collection of plasma for biochemistry. The femoral head of patients referred to hip replacement surgery due to coxarthrosis was removed during surgery and immediately transferred to the laboratory for tissue and cellular analyses in +4°C refrigerated shipping conditions to preserve the anatomical structure and bio‐functions as we previously described (Dang et al., 2015; Spinetti et al., 2013). Only material that would be discarded was collected for this study. BM cells were isolated from the femoral head by flushing with PBS (SIGMA COD.D8537) and filtering on a 70‐μm cell strainer (BD COD.AA352350). Both PB‐mononuclear cells (MNCs) and BM‐MNCs were isolated by gradient centrifugation using Histopaque Ficoll (SIGMA COD.1077). PB‐MNCs and BM‐MNCs were assessed for viability by Trypan Blue (SIGMA COD.T8154), and an average of 50 × 10^7^ cells was immunomagnetically sorted using Diamond CD34^+^ HSPC Isolation Kit Human (MILTENYI COD.130‐094‐531). The purity of selected cells was assessed by flow cytometry.

### 4.7 Flow cytometry

Stem/progenitor cells from PB were measured as previously described in detail (Fadini et al., 2006). After red blood cell lysis, cells were stained with anti‐CD34 (Becton Dickinson), CD133 (Miltenyi Biotec), and CD45 (BD BioscencesTM) monoclonal antibodies. HSPCs were defined as CD34^+^, CD133^+^, CD34^+^CD133^+^, and CD34^+^CD45^dim^ cells. At least 5–10 × 10^6^ events were acquired. We considered both relative (positive events/10^6^ events) and absolute cell counts. Absolute levels were obtained by multiplying relative levels to white blood cells (/ml). HSPCs levels were quantified by the same operator using the same method and materials throughout the study.

### 4.8 Immunohistochemistry

BM samples (~1 cm^3^) were harvested, fixed, and embedded as previously described (Dang et al., 2015; Spinetti et al., 2013). For immunohistochemical staining, 3 μm BM slides were automatically processed and stained with a BenchMark ULTRA slide stainer (Ventana).

### 4.9 Transcription profiling

Total RNA was extracted from 0.5–2 × 10^5^ purified CD34^+^HSPCs by using the Arcturus PicoPure (Applied BioSystem). The extracted RNA was used for NGS librarie preparation following Illumina instruction (TruSeq Stranded Total RNA Library Prep Gold), while sequencing was performed on NovaSeqTM 6000 (Illumina) running in a 50‐bp pair‐end mode. After quality control was performed with FASTQC, reads were mapped on the human genome (GRCh37/hg19) using Tophat/Bowtie2. Raw gene expression values were obtained with HTseq and used to perform PCA through the package. Only 10% of most variable genes were included. HTseq counts were also used to measure the differential gene expression with the edgeR package, applying the TMM normalization. Genes were identified as differentially expressed (DEGs) when the following criteria were met: UP DEGs = log2Fc ≥1, false discovery rate (FDR) ≤0.1, RPKM >1; DOWN DEGs = log2Fc ≤ −1, false discovery rate (FDR) ≤0.1, and RPKM >1. Heatmaps were generated using R package gplots. The Euclidean method was chosen to measure the distance among the samples. Values were centered and scaled in the row direction using the option inside the heatmap function. The heatmap of all expressed genes was drawn taking into account genes with a TPM >0 in at least one sample. Ingenuity pathway analysis was run on DEGs and biological functions with a *z* score superior to 1.5 in BM analysis and more than 3.5 in PB analysis were selected.

### 4.10 Quantitative analyses of soluble factors

The levels of cytokines, chemokines, and growth factors were determined in 40 µl cleared plasma duplicates by using the multi‐analyte immunoassay kit Human ProcartaPlex Panel 1, ThermoFisher through a MAGPIX Luminex instrument (Luminex Corporation). The absolute concentration for each molecule was obtained according to the manufacturer’s instructions upon comparison with standard curves in the linear range. Heatmaps were generated using Morpheus software from Broad Institute (https://software.broadinstitute.org/morpheus/). Functional network protein analysis was carried out using STRING version 11.0 (https://version‐11‐0.string‐db.org). In addition, a commercial ELISA kit was used to test the plasma levels of S100A8/A9 (EKMRP8/14 Bühlmann Laboratories Ag, Switzerland), following the manufacturer’s instructions. Plasma immunoreactive levels of S100A8/A9 showed a skewed distribution, requiring log transformation for normalization.

### 4.11 Statistics

Descriptive statistics included proportions for categorical and mean (standard deviation) or median (IQR) for continuous variables, according to the data distribution. Normality in the data distribution was checked using the Kolmogorov–Smirnov test. The median value of frailty indexes was used to classify patients into two groups (below/above the median). Comparison between groups was performed using two‐tailed unpaired Student's t test for continuous variables and the chi‐squared test (or Fisher's exact test) for categorical variables. Non‐normally distributed continuous variables were log‐transformed to be included in the analysis using parametric tests. If this was not possible, a non‐parametric test (Wilcoxon signed‐rank) was applied. A univariate linear regression analysis was performed to assess the relationship between frailty and HSPC and S100A8/A9 levels, followed by multivariate linear regression models to adjust each significant relationship for relevant confounders. The Cox proportional hazards model was used to determine whether frailty and HSPC and S100A8/A9 levels were predictors of clinical outcomes at 1 year of follow‐up, adjusted for relevant confounders. Confounders were selected through statistical significance observed in the univariate Cox model. Spearman partial correlation coefficient was used to assess the association between PF and variables recorded at the enrollment, in order to adjust for age and sex effect. 95% confidence intervals for correlation coefficients were estimated by using Fisher’s Z transformation. Similarly, Pearson partial correlation coefficient was used to evaluate the association between IFi and baseline variables. Statistical significance was accepted at *p* < 0.05, and all tests were two‐tailed. Statistical analyses were performed using the SPSS statistics version 22.0 (IBM Corp.) and SAS software version 9.4 (SAS Institute).

## CONFLICT OF INTEREST

The authors have declared that no competing interest exists.

## AUTHOR CONTRIBUTIONS

B.B.M, P.M.T., V.R., C.O., S.F., P.G., C.F., C.R., and R.A. conducted experiments and acquired data. M.F. analyzed data. T.G., A.P., O.P, C.F., B.A., and DS.S. contributed to study design and data acquisition. F.G.P., G.M., M.P., and S.G. designed the research studies, acquired funding, and wrote the manuscripts. Co‐first authorship order was assigned based on the involvement in the original study design and data analysis performed.

## ETHICS APPROVAL

The study was conducted following the Declaration of Helsinki and under the ethical approval of the internal ethical Committees of the IRCCS MultiMedica and the University Hospital of Padova.

## PATIENT CONSENT STATEMENT

All patients provided written informed consent to the study.

## Supporting information

Supplementary Material

## Data Availability

RNA‐seq data are deposited on GEO repository and accessible with GSE190446 number. All the data that support the figures and the other findings are available upon request to the corresponding authors.
