# Supplementary material for: Hematopoietic progenitor cell liabilities and alarmins S100A8/A9‐related inflammaging associate with frailty and predict poor cardiovascular outcomes in older adults
Source: Aging Cell. 2022 Feb 15;21(3):e13545. doi: 10.1111/acel.13545 (PMC8920446; doi:10.1111/acel.13545)
Supplement: Supplementary file 1 — Supplementary Material [file ACEL-21-e13545-s001.docx]

**Online-only Supplements**

**Title: Hematopoietic progenitor cell liabilities and alarmins S100A8/A9 – related inflammaging associate with frailty and predict poor cardiovascular outcomes in older adults**

**Authors:** Benedetta Maria Bonora, Maria Teresa Palano, Gianluca Testa, Gian Paolo Fadini, Elena Sangalli, Fabiana Madotto, Giuseppe Persico, Francesca Casciaro, Rosa Vono, Ornella Colpani, Francesco Scavello, Roberta Cappellari, Pasquale Abete, Patrizia Orlando, Franco Carnelli, Andrea Giovanni Berardi, Stefano De Servi, Angela Raucci, Marco Giorgio, Paolo Madeddu, and Gaia Spinetti

**Online-Only Figures**

**Figure S1**


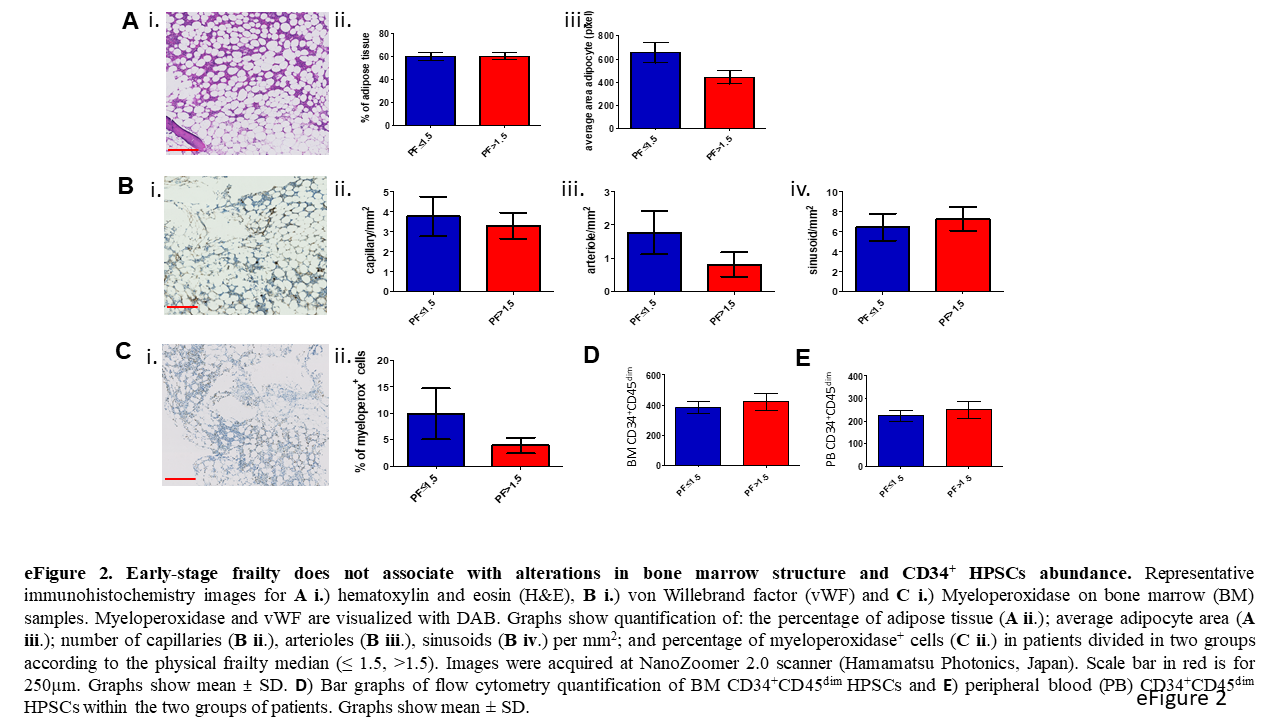


**Figure S1. Early-stage frailty does not associate with alterations in bone marrow structure and CD34^+^ HPSCs abundance.** Representative immunohistochemistry images for **A i.**) hematoxylin and eosin (H&E), **B i.**) von Willebrand factor (vWF), and **C i.**) Myeloperoxidase on bone marrow (BM) samples. Myeloperoxidase and vWF are visualized with DAB. Graphs show quantification of the percentage of adipose tissue (**A ii**.); average adipocyte area (**A iii**.); number of capillaries (**B ii**.), arterioles (**B iii**.), sinusoids (**B iv**.) per mm^2^; and percentage of myeloperoxidase^+^ cells (**C ii**.) in patients divided into two groups according to the physical frailty median (≤ 1.5, >1.5). Images were acquired at NanoZoomer 2.0 scanner (Hamamatsu Photonics, Japan). The scale bar in red is for 250µm. Graphs show mean ± SD. **D**) Bar graphs of flow cytometry quantification of BM CD34^+^CD45^dim^ HPSCs and **E**) peripheral blood (PB) CD34^+^CD45^dim^ HPSCs within the two groups of patients. Graphs show mean ± SD.

**Figure S2**


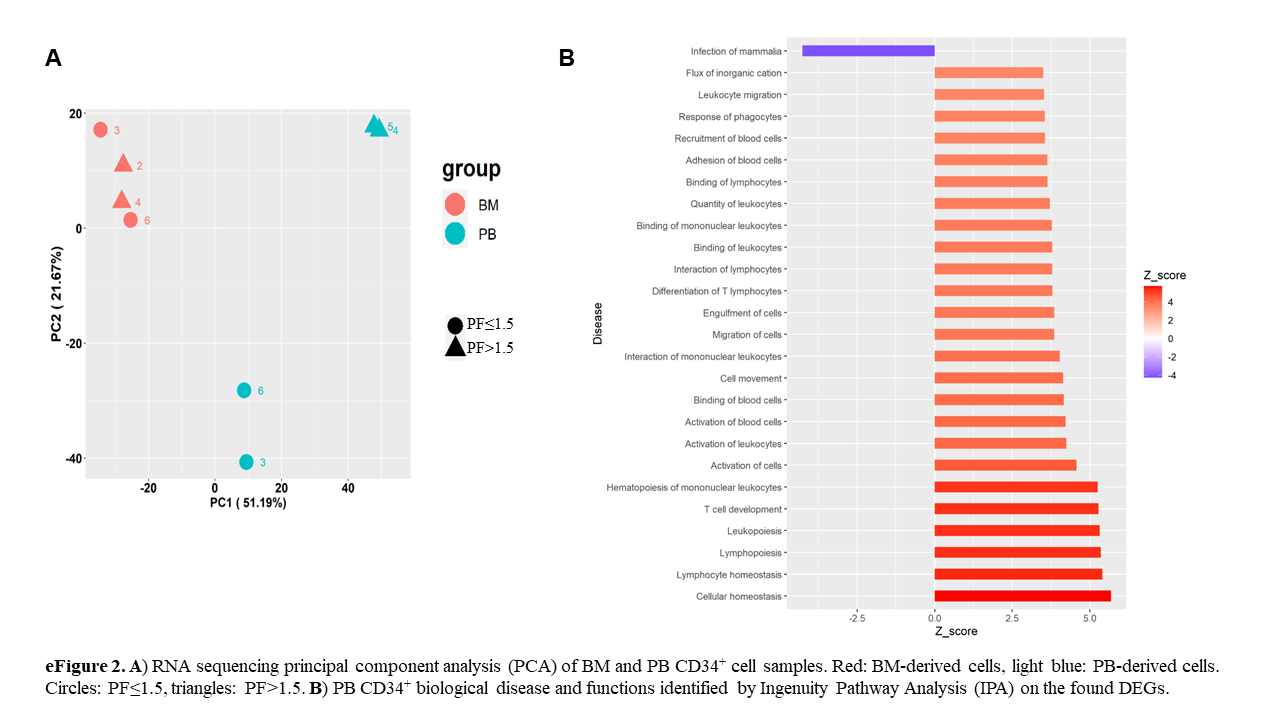


**Figure S2. A**) RNA sequencing principal component analysis (PCA) of BM and PB CD34^+^ cell samples. Red: BM-derived cells, light blue: PB-derived cells. Circles: PF≤1.5, triangles: PF>1.5. **B**) PB CD34^+^ biological disease and functions identified by Ingenuity Pathway Analysis (IPA) on the found DEGs.

**Figure S3**

**
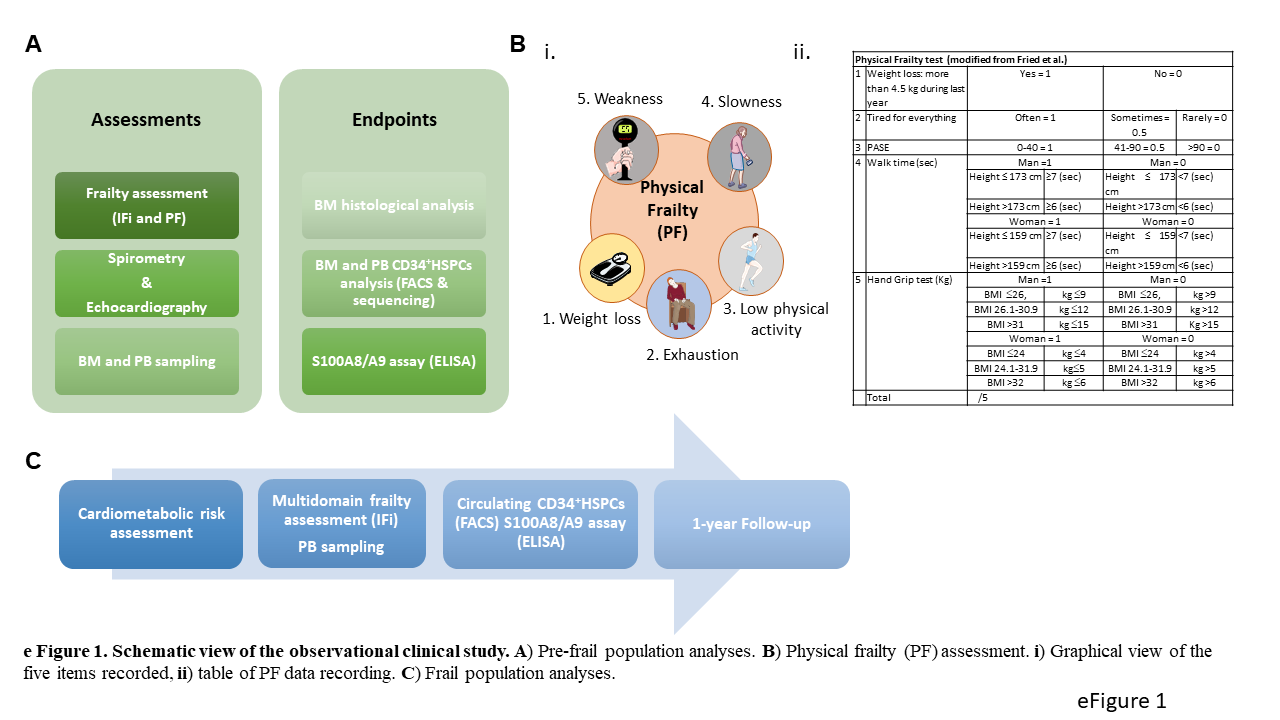
**

**Figure S3. Schematic view of the observational clinical study. A**) Pre-frail population analyses. **B**) Physical frailty (PF) assessment. **i**) Graphical view of the five items recorded, **ii**) table of PF data recording. **C**) Frail population analyses.

**Online-Only Tables**

**Table S1. Pre frail cohort. Laboratory tests.**

|  | **Pre-frail cohort**  **(N=35)** |
| --- | --- |
| **Complete blood counts** |  |
| White blood cells (×10^3^/uL) | 6.52 ± 1.38 |
| Red blood cells (×10^3^/uL) | 4.67 ± 0.54 |
| Hemoglobin (gr/dL) | 14.21 ± 1.64 |
| Hematocrit (%) | 44.51 ± 4.77 |
| Mean corpuscular volume (fl) | 95.43 ± 5.03 |
| Platelets (×10^3^/uL) | 246.74 ± 52.31 |
| Neutrophils (×10^3^/uL) | 3.92 ± 1.09 |
| Lymphocytes (×10^3^/uL) | 1.78 ± 0.48 |
| Monocytes (×10^3^/uL) | 0.60 ± 0.15 |
| Eosinophils (×10^3^/uL) | 0.18 ± 0.16 |
| Basophils (×10^3^/uL) | 0.04 ± 0.02 |
| Neutrophils / Lymphocytes | 2.33 ± 0.84 |
| **Biochemical tests** |  |
| Thyroid-stimulating hormone(uUI/ml) | 1.78 ± 1.03 |
| Free thyroxine TF4 (ng/dl) | 1.23 ± 0.33 |
| Free triiodothyronine TF3 (ng/dl) | 3.26 ± 0.47 |
| Urea (mg/dl)* | 46.04 ± 17.61 |
| Creatinine (mg/dl)° | 0.95 ± 0.29 |
| Urate (mg/dl) | 5.89 ± 1.62 |
| Glucose (mg/dl)° | 102.94 ± 14.79 |
| Bilirubin (mg/dl) | 0.85 ± 0.37 |
| Aspartate transaminase (U/L)° | 23.36 ± 7.26 |
| Alanine transaminase (U/L)° | 22.27 ± 13.44 |
| γ-glutamyltransferase  (U/L) | 39.06 ± 45.91 |
| Sodium (mEq/L)° | 142.61 ± 3.10 |
| Potassium (mEq/L)° | 4.48 ± 0.46 |
| Calcium (mmol/L) | 2.41 ± 0.15 |
| Inorganic phosphate (mmlo/L) | 1.13 ± 0.17 |
| Total cholesterol (mg/dl) | 200.63 ± 34.82 |
| HDL cholesterol (mg/dl) | 56.40 ± 14.58 |
| LDL cholesterol (mg/dl) | 119.09 ± 30.07 |
| Triglycerides (mg/dl) | 125.63 ± 51.10 |
| Lactate dehydrogenase (U/L) | 200.60 ± 35.61 |
| Creatine phosphokinase (U/L)° | 108.91 ± 56.07 |
| Creatine kinase CK-MB (ng/ml) | 2.30 ± 1.33 |
| CK-MB ratio (%)^ | 2.34 ± 1.35 |
| Total proteins (g/dl) | 7.01 ± 0.45 |
| Albumin (g/dl) | 4.09 ± 0.29 |
| Alpha 1 globulins (g/dl-%) | 0.28 ± 0.05 |
| Alpha 2 globulins (g/dl-%) | 0.75 ± 0.11 |
| Beta 1 globulins (g/dl-%) | 0.44 ± 0.06 |
| Beta 2 globulins (g/dl-%) | 0.39 ± 0.07 |
| Gamma globulins (g/dl-%) | 1.07 ± 0.23 |
| C-reactive protein (mg/dL)^†^ | 0.32 ± 0.29 |
| B-type natriuretic peptide (pg/ml) | 74.11 ± 78.42 |
| International normalized ratio° | 1.11 ± 0.44 |
| Activated partial thromboplastin time (seconds)° | 26.48 ± 3.79 |
| Fibrinogen (mg/dl) | 356.06 ± 94.75 |
| Bone-specific alkaline phosphatase (µg/L)^‡^ | 19.98 ± 7.26 |
| Carboxy-terminal collagen crosslinks (ng/ml)° | 0.44 ± 0.41 |

*Values are mean ± standard deviation. Abbreviations. HDL= high density lipoprotein; LDL= low density lipoprotein; CK-MB= creatine kinase (isoform of heart muscle). * Data was available for 26 subjects (74.3%). ° Data was available for 33 subjects (94.3%). ^ Data was available for 31 subjects (88.6%). † Data was available for 32 subjects (91.4%). ‡ Data was available for 34 subjects (97.1%).*

**Table S2. Pre frail cohort. Heart structure/function and blood pressure.**

|  | **Pre-frail cohort**  **(N=35)** |
| --- | --- |
| **Echocardiography - Left ventricle** |  |
| Interventricular septum thickness (mm)* | 11.16 ± 1.86 |
| Posterior wall thickness (mm) | 10.12 ± 1.34 |
| End-diastolic diameter (mm) | 42.73 ± 5.94 |
| End-diastolic volume (ml) | 98.02 ± 26.27 |
| End-systolic volume (ml) | 37.83 ± 16.81 |
| Ejection fraction (%) | 62.09 ± 7.09 |
| Relative wall thickness | 0.48 ± 0.09 |
| **Echocardiography - Right ventricle** |  |
| End-diastolic diameter (mm) | 28.61 ± 3.63 |
| TAPSE (mm) | 23.66 ± 4.26 |
| **Echocardiography – Tricuspid** |  |
| Systolic ventricular-atrial gradient (mmHg)° | 22.70 ± 4.51 |
| Pulmonary arterial systolic pressure (mmHg)^ | 27.85 ± 4.45 |
| **Echocardiography - Diastolic function** |  |
| E mitral wave (cm/sec)* | 65.34 ± 14.42 |
| A mitral wave (cm/sec)^†^ | 79.55 ± 19.09 |
| E/A mitral ratio^†^ | 0.83 ± 0.22 |
| Deceleration time (msec)^‡^ | 265.54 ± 58.27 |
| Septal E (cm/sec)^§^ | 6.38 ± 1.80 |
| Lateral E (cm/sec)^§^ | 8.39 ± 3.07 |
| E mitral/septal E ^#^ | 10.82 ± 2.69 |
| E mitral/lateral E ^#^ | 8.67 ± 3.51 |
| E mitral/average E ^#^ | 9.77 ± 2.65 |
| **Echocardiography - Left atrium** |  |
| Diameter (mm)* | 37.53 ± 5.80 |
| Area 4C (cm^2^)^‡^ | 19.79 ± 4.71 |
| Volume (ml)^&^ | 62.03 ± 21.32 |
| **Echocardiography – Aorta** |  |
| Diameter-root (mm) | 33.18 ± 3.89 |
| Cusp Separation (mm)^#^ | 15.66 ± 3.50 |

*Values are mean ± standard deviation. Abbreviations. TAPSE=tricuspid annular plane excursion. * Data was available for 34 subjects (97.1%). ° Data was available for 27 subjects (77.1%). ^ Data was available for 26 subjects (74.3%). † Data was available for 31 subjects (88.6%). ‡ Data was available for 24 subjects (68.6%). § Data was available for 33 subjects (94.3%). # Data was available for 32 subjects (91.4%). & Data was available for 20 subjects (57.1%).*

**Table S3. Pre frail cohort. Pulmonary function assessment by spirometry.**

|  | **Pre-frail cohort**  **(N=35)** |
| --- | --- |
| FVC (L) | 2.97 ± 0.82 |
| FEV1 (L) | 2.26 ± 0.62 |
| FEV1 /FVC (%) | 76.34 ± 7.98 |
| PEF (L/s) | 5.79 ± 1.83 |
| VC max (L)* | 2.97 ± 0.74 |

*Values are mean ± standard deviation. Abbreviations. FEV1= forced expiratory volume (at the timed interval of 1 sec); FVC= forced vital capacity; PEF= peak expiratory flow; VC= vital capacity. * Data was available for 30 subjects (85.7%).*

**Table S4. Pre frail cohort. Clinical characteristics of pre-frail patients stratified according to the median value of multidomain frailty (Italian Frailty index, IFi ≤ 9, >9).**

|  | **IFi ≤ 9** | **IFi > 9** | **p-value** |
| --- | --- | --- | --- |
|  | **(N=18)** | **(N=17)** |  |
| Age (years), mean  ± SD | 73.0 ± 6.6 | 77.12 ± 4.03 | 0.0523 |
| Males, n (%) | 13 (72.2) | 7 (41.2) | 0.0636 |
| Body Mass Index (kg/m^2^), mean ± SD | 27.0 ± 3.7 | 28.9 ± 5.3 | 0.2351 |
| **Spirometry** |  |  |  |
| FVC (L), mean  ± SD | 3.5 ± 0.7 | 2.4 ± 0.6 | <.0001 |
| FEV1 (L), mean  ± SD | 2.6 ± 0.6 | 1.9 ± 0.4 | <.0001 |
| FEV1 /FVC (%), mean  ± SD | 75.4 ± 6.3 | 77.3 ± 9.5 | 0.5029 |
| PEF (L/s), mean  ± SD | 6.9 ± 1.3 | 4.6 ± 1.5 | <.0001 |
| VC max (L)*, mean  ± SD | 3.4 ± 0.6 | 2.6 ± 0.6 | 0.0014 |
| **Echocardiography** |  |  |  |
| Pulmonary arterial pressure (mmHg)*, mean ± SD | 27.0 ± 4.0 | 28.8 ± 4.9 | 0.3133 |
| *Left ventricle* |  |  |  |
| Relative wall thickness, mean ± SD | 0.5 ± 0.1 | 0.5 ± 0.1 | 0.0248 |
| *Diastolic function* |  |  |  |
| A mitral wave (cm/sec)^°^, mean ± SD | 71.1 ± 15.7 | 88.6 ± 18.7 | 0.0082 |
| E/A mitral ratio^°^, mean ± SD | 0.9 ± 0.3 | 0.7 ± 0.2 | 0.0328 |
| Septal E (cm/sec)^^^, mean ± SD | 10.0 ± 2.8 | 11.8 ± 2.3 | 0.0505 |
| **Blood pressure** |  |  |  |
| Max (mmHg), mean ± SD | 134.2 ± 18.1 | 146.5 ± 18.1 | 0.0526 |
| Average (mmHg), mean ± SD | 97.5 ± 8.4 | 104.7 ± 10.1 | 0.0290 |
| **Frailty variables** |  |  |  |
| Physical Frailty |  |  |  |
| Mean ± SD | 0.9 ± 0.8 | 2.1 ± 1.0 | 0.0008 |
| Median [IQR] | 0.8 [0.5-1.5] | 2.0 [1.5-3.0] |  |
| Mini nutritional assessment, median [IQR] | 28.5 [27.0-29.0] | 27.0 [25.5-28.5] | 0.0364 |
| Cumulative Illness Rating Scale, median [IQR] | 1.4 ± 0.1 | 1.6 ± 0.3 | 0.0612 |
| Tinetti scale, median [IQR] | 25.5 [24.0-27.0] | 20.0 [16.0-22.0] | 0.0007 |
| 6 minutes walking test (m)^†^, mean ± SD | 363.0 ± 105.1 | 256.9 ± 130.5 | 0.0149 |

*Abbreviations. IQR= interquartile range [1st quartile-3rd quartile]; SD= standard deviation.*

** For 9 subjects (5 with IFi ≤ 9, 4 IFi > 9), data was not available.*

*° For 4 subjects (2 with IFi ≤ 9, 2 IFi > 9), data was not available.*

*^ For 3 subjects (1 with IFi ≤ 9, 2 IFi > 9), data was not available.*

*† For 7 subjects (4 with IFi ≤ 9, 3 IFi > 9), data was not available.*

*Note 1. For discrete variables, comparisons between groups were performed using the Chi-square test.*

*Note 2. For continuous variables, comparisons between groups were performed using Student’s T-test or Wilcoxon–Mann–Whitney test according to Normal data distribution.*

**Table S5. Pre frail cohort. Clinical characteristics of pre-frail patients stratified according to the median physical frailty phenotype (PF) value.**

|  | **PF ≤ 1.5** | **PF > 1.5** | **p-value** |
| --- | --- | --- | --- |
|  | **(N=22)** | **(N=13)** |  |
| Age (years), mean  ± SD | 74.4 ± 6.2 | 76.0 ± 5.2 | 0.5255 |
| Males, n (%) | 15 (68.2) | 5 (38.5) | 0.0860 |
| Body Mass Index (kg/m^2^), mean ± SD | 28.1 ± 3.7 | 27.5 ± 6.0 | 0.7640 |
| **Spirometry** |  |  |  |
| FVC (L), mean  ± SD | 3.3 ± 0.7 | 2.5 ± 0.8 | 0.0025 |
| FEV1 (L), mean  ± SD | 2.5 ± 0.5 | 1.83 ± 0.5 | 0.0008 |
| FEV1 /FVC (%), mean  ± SD | 76.8 ± 7.5 | 75.6 ± 9.0 | 0.6874 |
| PEF (L/s), mean  ± SD | 6.6 ± 1.7 | 4.4 ± 1.1 | <.0001 |
| VC max (L)*, mean  ± SD | 3.2 ± 0.6 | 2.5 ± 0.8 | 0.0230 |
| **Echocardiography** |  |  |  |
| Pulmonary arterial pressure (mmHg)°, mean ± SD | 27.4 ± 4.4 | 28.4 ± 4.6 | 0.5560 |
| *Left ventricle* |  |  |  |
| Relative wall thickness, mean ± SD | 0.5 ± 0.1 | 0.5 ± 0.1 | 0.4423 |
| *Diastolic function* |  |  |  |
| A mitral wave (cm/sec)^°^, mean ± SD | 75.5 ± 17.6 | 86.8 ± 20.3 | 0.1168 |
| E/A mitral ratio^°^, mean ± SD | 0.9 ± 0.3 | 0.7 ± 0.1 | 0.1054 |
| Septal E (cm/sec)^^^, mean ± SD | 10.5 ± 2.5 | 11.3 ± 3.1 | 0.4307 |
| **Blood pressure** |  |  |  |
| Max (mmHg), mean ± SD |  | 143.46 ± 25.77 | 0.5037 |
| Average (mmHg), mean ± SD | 100.00 ± 7.05 | 102.69 ± 13.46 | 0.5132 |
| **Frailty variables** |  |  |  |
| Italian Frailty index |  |  |  |
| Mean ± SD | 7.4 ± 3.9 | 10.9 ± 2.7 | 0.0031 |
| Median [IQR] | 6.1 [4.5 - 11.0] | 10.5 [10.0 - 13.5] |  |
| Mini nutritional assessment, median [IQR] | 28.3 [27.0 - 29.0] | 27.0 [25.0 - 28.5] | 0.0332 |
| Cumulative Illness Rating Scale, median [IQR] | 1.4 [1.3 - 1.6] | 1.6 [1.5 - 1.8] | 0.0112 |
| Tinetti scale, median [IQR] | 24.0 [21.0 - 26.0] | 19.0 [16.0 - 22.0] | 0.0074 |
| 6 minutes walking test (m)^, mean ± SD | 374.9 ± 95.7 | 199.8 ± 99.1 | <.0001 |

*Abbreviation. IQR= interquartile range [1st quartile-3rd quartile]; FEV1= forced expiratory volume (at timed interval of 1 sec); FVC= forced vital capacity; PEF= peak expiratory flow; SD= standard deviation; VC= vital capacity.*

** For 5 subjects (3 with physical frailty ≤ 1.5, 2 with physical frailty > 1.5), data was not available.*

*° For 9 subjects (8 with physical frailty ≤ 1.5, 1 with physical frailty > 1.5), data was not available.*

*^ For 1 subject with physical frailty > 1.5, data was not available.*

*Note 1. For discrete variables, comparisons between groups were performed using the Chi-square test.*

*Note 2. For continuous variables, comparisons between groups were performed using Student’s T-test or Wilcoxon–Mann–Whitney test according to Normal data distribution.*

**Table S6. Pre frail cohort. Association between Physical Frailty phenotype and patients’ characteristics (Spearman partial correlation coefficients according to age and sex).**

|  | **Correlation coefficient**  **(95% CI)** | **p-value** |
| --- | --- | --- |
| Weight (kg) | -0.085 (-0.416 ; 0.266) | 0.6397 |
| Height (m) | -0.306 (-0.587 ; 0.042) | 0.0837 |
| Body Mass Index (kg/m^2^) | 0.011 (-0.333 ; 0.353) | 0.9511 |
| **Echocardiography – Diastolic function** |  |  |
| E/A ratio* | -0.401 (-0.669 ; -0.040) | 0.0304 |
| **Spirometry** |  |  |
| FVC (L) | -0.592 (-0.778 ; -0.312) | 0.0002 |
| FEV1 (L) | -0.547 (-0.749 ; -0.250) | 0.0008 |
| FEV1 /FVC (%) | -0.106 (-0.434 ; 0.246) | 0.5591 |
| PEF (L/s) | -0.618 (-0.793 ; -0.349) | <.0001 |
| VC max (L)° | -0.463 (-0.713 ; -0.108) | 0.0123 |
| **Frailty variables** |  |  |
| Physical activity scale for the elderly | -0.651 (-0.813 ; -0.397) | <.0001 |
| Cumulative Illness Rating Scale | 0.461 (0.140 ; 0.694) | 0.0063 |
| Tinetti scale | -0.514 (-0.728 ; -0.207) | 0.0019 |
| 6 minutes walking test (m)^ | -0.503 (-0.725 ; -0.187) | 0.0029 |
| Time spent for 4 meters (s)^ | 0.499 (0.182 ; 0.7223) | 0.0031 |
| **Circulating factors** |  |  |
| S100A8/A9^ | 0.470 (0.145 ; 0.703) | 0.0061 |
| EGF^†^ | 0.437 (0.0.51 ; 0.710) | 0.0279 |
| Fractalkine^‡^ | 0.501 (0.140 ; 0.744) | 0.0083 |
| PDL-1^†^ | 0.504 (0.136 ; 0.750) | 0.0093 |
| RANTES^‡^ | 0.406 (0.023 0.686) | 0.0386 |
| TNF-α^‡^ | 0.406 (0.022 ; 0.688) | 0.0388 |

*Abbreviations. CI= confidence interval; FEV1= forced expiratory volume (at timed interval of 1 sec); FVC= forced vital capacity; PEF= peak expiratory flow; VC= vital capacity. * Data was available for 31 subjects (88.6%). ° Data was available for 30 subjects (85.7%). ^ Data was available for 34 subjects (97.1%). † Data was available for 27 subjects (77.1%). ‡ Data was available for 28 subjects (80.0%).*

**Table S7. Pre-frail cohort. Deregulated genes in bone marrow CD34+ cells.**

|  | **log_2_Fold Change** | **False Discovery Rate** |
| --- | --- | --- |
| **Up-regulated Gene** |  |  |
| ARG1 | 4.0200 | 0.0153 |
| C19orf51 | 5.3861 | 0.0000 |
| KLHL31 | 2.3525 | 0.0598 |
| LOC100288778 | 3.3142 | 0.0681 |
| LPAR6 | 1.9663 | 0.0821 |
| MAF | 2.3629 | 0.0614 |
| MKRN3 | 5.9425 | 0.0000 |
| MMP8 | 3.6575 | 0.0735 |
| NLRP2 | 3.7252 | 0.0735 |
| RETN | 5.0194 | 0.0290 |
| S100A8 | 3.5583 | 0.0290 |
| S100A9 | 2.8383 | 0.0648 |
| TRIM78P | 3.8843 | 0.0290 |
| ZNF702P | 2.3189 | 0.0511 |
| **Down-regulated Gene** |  |  |
| BGN | -4.5436 | 0.0066 |
| CHI3L2 | -4.7798 | 0.0170 |
| DCN | -6.8696 | 0.0318 |
| DKFZP564C196 | -4.0176 | 0.0247 |
| FOS | -2.0260 | 0.0290 |
| LOC284454 | -2.0685 | 0.0442 |

**Table S8. Pre-frail cohort. Top-20 deregulated genes in peripheral blood CD34+ cells.**

|  | **log_2_Fold Change** | **False Discovery Rate** |
| --- | --- | --- |
| **Up-regulated Gene** |  |  |
| LYZ | 5.1719 | 0.0181 |
| S100A9 | 7.6752 | 0.0011 |
| S100A8 | 8.2288 | 0.0007 |
| FCN1 | 5.8597 | 0.0104 |
| IFI30 | 4.1701 | 0.0827 |
| VCAN | 9.7287 | 0.0001 |
| CD14 | 10.3189 | 0.0001 |
| FPR1 | 7.9578 | 0.0056 |
| TUBB1 | 4.9528 | 0.0248 |
| MNDA | 5.1995 | 0.0264 |
| F13A1 | 4.9316 | 0.0416 |
| FGR | 4.3811 | 0.0576 |
| THBS1 | 5.3940 | 0.0296 |
| IL7R | 7.0763 | 0.0090 |
| S100A12 | 10.7715 | 0.0001 |
| TUBA4A | 4.6652 | 0.0368 |
| LCK | 4.6688 | 0.0364 |
| CLEC7A | 4.3571 | 0.0689 |
| LGALS3 | 6.3596 | 0.0047 |
| SLC2A3 | 4.6564 | 0.0458 |
| **Down-regulated Gene** |  |  |
| SCARB1 | -5.0461 | 0.0267 |
| RPS28 | -4.3515 | 0.0689 |
| ZBTB8A | -4.3500 | 0.0780 |
| EFHC2 | -6.2229 | 0.0104 |
| ZNF551 | -4.8488 | 0.0401 |
| KIAA1797 | -5.6271 | 0.0267 |
| EFNA1 | -7.7491 | 0.0040 |
| NMNAT3 | -6.7982 | 0.0055 |
| TEKT2 | -11.2090 | 0.0043 |
| GATM | -8.0865 | 0.0076 |
| LEPRE1 | -6.4898 | 0.0121 |
| CLEC9A | -8.8619 | 0.0056 |
| LOC730102 | -7.3348 | 0.0327 |
| DNASE1L3 | -5.8180 | 0.0605 |
| PLAU | -8.7690 | 0.0057 |
| PLS3 | -5.6329 | 0.0826 |
| KDELC1 | -10.8228 | 0.0017 |
| THNSL1 | -6.0259 | 0.0246 |
| CALN1 | -5.5187 | 0.0233 |
| PGM5 | -10.7401 | 0.0075 |

**Table S9. Frail cohort. Characteristics of frail patients stratify according to the median Italian frailty index (IFi) value.**

|  | **IFi ≤ 14.1**  **(N = 52)** | **IFi > 14.1**  **(N = 52)** | **p-value** |
| --- | --- | --- | --- |
| Age (years), mean ± SD | 78.7 ± 5.1 | 82.5 ± 6.1 | 0.001 |
| Males, n (%) | 26 (50.0) | 31 (59.6) | 0.431 |
| Body Mass Index (kg/m^2^) | 26.0 ± 4.0 | 27.6 ± 5.2 | 0.067 |
| Smoking habit, n (%) | 4 (7.7) | 2 (3.8) | 0.678 |
| **Chronic disease** |  |  |  |
| Charlson Comorbidity Index, mean ± SD | 6.6 ± 1.6 | 8.5 ± 2.3 | <0.001 |
| Diabetes, n (%) | 46 (88.5) | 41 (78.8) | 0.289 |
| Hypertension, n (%) | 46 (88.5) | 49 (94.2) | 0.488 |
| Coronary Artery Disease, n (%) | 12 (23.1) | 18 (34.6) | 0.279 |
| Cancer, n (%) | 7 (13.5) | 13 (25.0) | 0.213 |
| Osteoporosis, n (%) | 6 (11.5) | 8 (15.4) | 0.775 |
| Cerebral vascular disease, n (%) | 27 (51.9) | 20 (38.5) | 0.237 |
| Stroke or transient ischemic attack, n (%) | 4 (7.7) | 7 (13.5) | 0.526 |
| Chronic kidney disease, n (%) | 17 (32.7) | 22 (42.3) | 0.418 |
| Surgery, n (%) | 29 (55.8) | 34 (65.4) | 0.160 |
| Chronic obstructive pulmonary disease, n (%) | 0 (0.0) | 11 (21.1) | <0.001 |
| **Stem-Progenitor cell** |  |  |  |
| S100A8/A9, median [IQR] | 752.9  [461.4-1088.7] | 866.0  [576.7-1648.3] | 0.045 |
| CD34^+^, mean ± SD | 309.9 ± 165.9 | 248.8 ± 99.6 | 0.025 |
| CD133^+^, mean ± SD | 186.0 ± 96.3 | 141.9 ± 67.7 | 0.008 |
| CD34^+^CD133^+^, mean ± SD | 106.2 ± 69.3 | 85.4 ± 45.2 | 0.073 |
| CD34^+^CD45^neg^, mean ± SD | 25.2 ± 20.9 | 23.4 ± 20.4 | 0.651 |
| CD34^+^CD45^dim^, mean ± SD | 257.8 ± 151.4 | 203.9 ± 90.8 | 0.030 |

*Abbreviations. IQR=interquartile range [1st quartile – 3rd quartile]; SD= standard deviation. Note. Progenitor cell counts were expressed per 106 events. S100A8/A9 was log-transformed.*

**Table S10. Frail cohort. Relationship between stem-progenitor cells and Italian Frailty Index adjusted for age and sex. Results of multivariable linear regression models.**

|  | **Beta (95% confidence interval)** | **p-value** |
| --- | --- | --- |
| **Model 1, dependent variable = CD34^+^** | | |
| Age (years) | -0.01 (-0.04 ; 0.03) | 0.659 |
| Sex (Male vs Female) | -0.16 (-0.55 ; 0.23) | 0.423 |
| **Italian Frailty Index** | -0.03 (-0.06 ; 0.00) | 0.046 |
| **Model 2, dependent variable = CD133^+^** | | |
| Age (years) | -0.02 (-0.05 ; 0.02) | 0.326 |
| Sex (Male vs Female) | -0.26 (-0.64 ; 0.12) | 0.179 |
| **Italian Frailty Index** | -0.04 (-0.07 ; -0.01) | 0.011 |
| **Model 3, dependent variable = CD34^+^CD45^dim^** | | |
| Age (years) | 0.01 (-0.04 ; 0.03) | 0.666 |
| Sex (Male vs Female) | -0.16 (-0.55 ; 0.23) | 0.410 |
| **Italian Frailty Index** | -0.03 (-0.06 ; 0.00) | 0.032 |

**Table S11. Frail cohort. Relationship between Italian Frailty Index and stem-progenitor cells, adjusted for age and sex. Results of multivariable linear regression models.**

|  | **Beta (95% confidence interval)** | **p-value** |
| --- | --- | --- |
| **Model 1, dependent variable = Italian Frailty Index** | | |
| Age (years) | 0.37 (0.16 ; 0.57) | 0.001 |
| Sex (Male vs Female) | -1.49 (-3.95 ; 0.97) | 0.233 |
| **S100A8/A9** | 1.82 (0.16 ; 3.48) | 0.032 |
| **CD34^+^ (x SD)** | -0.96 (-2.22 ; 0.30) | 0.132 |
| **Model 2, dependent variable = Italian Frailty Index** | | |
| Age (years) | 0.33 (0.13 ; 0.54) | 0.002 |
| Sex (Male vs Female) | -1.68 (-4.10 ; 0.73) | 0.170 |
| **S100A8/A9** | 2.07 (0.50 ; 3.65) | 0.011 |
| **CD133 (x SD)** | -1.62 (-2.83 ; -0.40) | 0.010 |
| **Model 3, dependent variable = Italian Frailty Index** | | |
| Age (years) | 0.36 (0.15 ; 0.57) | 0.001 |
| Sex (Male vs Female) | -1.51 (-3.96 ; 0.95) | 0.226 |
| **S100A8/A9** | 1.85 (0.22 ; 3.48) | 0.026 |
| **CD34^+^ CD45^dim^ (x SD)** | -1.13 (-2.37 ; 0.11) | 0.073 |

*Abbreviations. SD=standard deviation. Note. S100A8/A9 was log-transformed.*

**Table S12. Frail cohort. Relationship between patient’s characteristics and occurrence of adverse cardiovascular events (MACE) during follow-up. Results of univariate Cox regression proportional hazard models (dependent variable = occurrence of MACE).**

|  | **Hazard Ratio**  **(95% confidence interval)** | **p-value** |
| --- | --- | --- |
| **Demographics** |  |  |
| Age (years) | 1.07 (1.00 ; 1.14) | 0.048 |
| Body Mass Index (kg/m^2^) | 1.02 (0.94 ; 1.10) | 0.646 |
| Smoking habit (reference No) | 1.07 (0.73 ; 1.57) | 0.719 |
| **Chronic disease** |  |  |
| Charlson Comorbidity Index | 1.19 (1.02 ; 1.38) | 0.026 |
| Diabetes (reference No) | 0.73 (0.30 ; 1.80) | 0.495 |
| Hypertension (reference No) | 1.35 (0.32 ; 5.67) | 0.684 |
| Coronary Artery Disease (reference No) | 1.70 (0.81 ; 3.61) | 0.164 |
| Cancer (reference No) | 1.10 (0.45 ; 2.70) | 0.835 |
| Osteoporosis (reference No) | 1.44 (0.55 ; 3.78) | 0.456 |
| Stroke or transient ischemic attack (reference No) | 0.59 (0.14 ; 2.47) | 0.468 |
| Chronic kidney disease (reference No) | 1.73 (0.83 ; 3.58) | 0.143 |
| Surgery (reference No) | 1.05 (0.50 ; 2.22) | 0.900 |
| Chronic obstructive pulmonary disease (reference No) | 1.86 (0.71 ; 4.87) | 0.208 |
| **Frailty** |  |  |
| Italian Frailty index | 1.10 (1.04 ; 1.16) | 0.001 |
| **Pharmacological therapy** |  |  |
| Use of glucose-lowering medication (reference No) | 0.60 (0.27 ; 1.36) | 0.225 |
| Use of ACEi or ARB (reference No) | 0.69 (0.33 ; 1.47) | 0.337 |
| Use of other anti-hypertensive (reference No) | 0.95 (0.42 ; 2.15) | 0.906 |
| Use of Statin (reference No) | 0.84 (0.40 ; 1.74) | 0.630 |
| Use of Anti-platelet (reference No) | 1.21 (0.83 ; 1.76) | 0.318 |
| **Stem-progenitor cells** |  |  |
| S100A8/A9 | 1.76 (1.12 ; 2.78) | 0.015 |
| CD34^+^ | 0.61 (0.38 ; 0.95) | 0.030 |
| CD133^+^ | 0.53 (0.32 ; 0.85) | 0.009 |
| CD34^+^CD133^+^ | 0.49 (0.29 ; 0.81) | 0.006 |
| CD34^+^CD45^dim^ | 0.62 (0.40 ; 0.97) | 0.037 |

*Abbreviations. ACEi= Angiotensin-converting enzyme inhibitors ; ARB= Angiotensin receptor blockers. Note. S100A8/A9 was log-transformed.*

**Table S13. Frail cohort. Relationship between stem-progenitor cells and occurrence of adverse cardiovascular events (MACE) during follow-up, adjusted for confounders (age, Italian Frailty Index). Results of multivariable Cox regression proportional hazard models (dependent variable = occurrence of MACE).**

|  | **Hazard Ratio**  **(95% confidence interval)** | **p-value** |
| --- | --- | --- |
| **Model 1, dependent variable = occurrence of MACE** | | |
| Age (years) | 1.04 (0.97 - 1.11) | 0.266 |
| Italian Frailty Index | 1.07 (1.01 - 1.14) | 0.027 |
| **S100A8/A9** | 1.55 (0.93 - 2.60) | 0.095 |
| **CD34^+^ (x SD)** | 0.68 (0.42 - 1.21) | 0.131 |
| **Model 2, dependent variable = occurrence of MACE** | | |
| Age (years) | 1.04 (0.97 - 1.11) | 0.292 |
| Italian Frailty Index | 1.06 (1.00 - 1.13) | 0.045 |
| **S100A8/A9** | 1.81 (1.05 - 3.13) | 0.032 |
| **CD133^+^ (x SD)** | 0.65 (0.40 - 1.05) | 0.078 |
| **Model 3, dependent variable = occurrence of MACE** | | |
| Age (years) | 1.04 (0.97 - 1.12) | 0.235 |
| Italian Frailty Index | 1.07 (1.01 - 1.14) | 0.027 |
| **S100A8/A9** | 1.73 (1.00 - 2.98) | 0.050 |
| **CD34^+^CD133^+^(x SD)** | 0.53 (0.32 - 0.87) | 0.012 |
| **Model 4, dependent variable = occurrence of MACE** | | |
| Age (years) | 1.04 (0.97 - 1.11) | 0.262 |
| Italian Frailty Index | 1.07 (1.01 - 1.14) | 0.029 |
| **S100A8/A9** | 1.58 (0.95 - 2.63) | 0.081 |
| **CD34^+^CD45^dim^ (x SD)** | 0.69 (0.42 - 1.12) | 0.129 |

*Abbreviations. SD=standard deviation. Note. S100A8/A9 was log-transformed.*

**Table S14. Frail cohort. Predictors for mortality during follow-up period. Results of multivariable Cox regression proportional hazard model (dependent variable = dead).**

|  | **Hazard Ratio**  **(95% confidence interval)** | **p-value** |
| --- | --- | --- |
| Age (years) | 1.01 (0.92 - 1.17) | 0.808 |
| Italian Frailty Index | 1.11 (1.01 - 1.22) | 0.024 |
| Cancer | 2.69 (0.89 - 8.12) | 0.079 |
| S100A8/A9 | 2.28 (1.20 - 4.31) | 0.012 |

*Note. S100A8/A9 was log-transformed.*
